# P300 ERP System Utilizing Wireless Visual Stimulus Presentation Devices

**DOI:** 10.3390/s25123592

**Published:** 2025-06-07

**Authors:** Yuta Sasatake, Kojiro Matsushita

**Affiliations:** 1 Intelligent Production Technology Research & Development Center for Aerospace, Institute for Advanced Study, Gifu University, Gifu 501-1193, Japan; 2 Department of Mechanical Engineering, Gifu University, Gifu 501-1193, Japan; matsushita.kojiro.h7@f.gifu-u.ac.jp

**Keywords:** EEG, BCI, ERP-P300, baseline, wireless visual stimulation

## Abstract

The P300 event-related potential, evoked by attending to specific sensory stimuli, is utilized in non-invasive brain–computer interface (BCI) systems and is considered the only interface through which individuals with complete paralysis can operate devices based on their intention. Conventionally, visual stimuli used to elicit P300 have been presented using displays; however, placing a display directly in front of the user obstructs the field of view and prevents the user from perceiving their surrounding environment. Moreover, every time the user changes posture, the display must be repositioned accordingly, increasing the burden on caregivers. To address these issues, we propose a novel system that employs wirelessly controllable LED visual stimulus presentation devices distributed throughout the surrounding environment, rather than relying on traditional displays. The primary challenge in the proposed system is the communication delay associated with wireless control, which introduces errors in the timing of stimulus presentation—an essential factor for accurate P300 analysis. Therefore, it is necessary to evaluate how such delays affect P300 detection accuracy. The second challenge lies in the variability of visual stimulus strength due to differences in viewing distance caused by the spatial distribution of stimulus devices. This also requires the validation of its impact on P300 detection. In Experiment 1, we evaluated system performance in terms of wireless communication delay and confirmed an average delay of 352.1 ± 30.9 ms. In Experiment 2, we conducted P300 elicitation experiments using the wireless visual stimulus presentation device under conditions that allowed the precise measurement of stimulus presentation timing. We compared P300 waveforms across three conditions: (1) using the exact measured stimulus timing, (2) using the stimulus timing with a fixed compensation of 350 ms for the wireless delay, and (3) using the stimulus timing with both the 350 ms fixed delay compensation and an additional pseudo-random error value generated based on a normal distribution. The results demonstrated the effectiveness of the proposed delay compensation method in preserving P300 waveform integrity. In Experiment 3, a system performance verification test was conducted on 21 participants using a wireless visual presentation device. As a result, statistically significant differences (*p* < 0.01) in amplitude between target and non-target stimuli, along with medium or greater effect sizes (Cohen’s d: 0.49–0.61), were observed under all conditions with an averaging count of 10 or more. Notably, the P300 detection accuracy reached 85% with 40 averaging trials and 100% with 100 trials. These findings demonstrate that the system can function as a P300 speller and be utilized as an interface equivalent to conventional display-based methods.

## 1. Introduction

In recent years, research on human–machine interfaces (HMIs) utilizing biosignals has advanced in the medical and welfare fields [1,2]. In particular, brain–computer interfaces (BCIs) based on electroencephalogram (EEG) signals have attracted attention as an essential assistive technology, as they enable individuals with severe physical impairments or complete paralysis—such as those with amyotrophic lateral sclerosis (ALS), brainstem infarction, cerebral palsy, or spinal cord injuries—to operate computers, often as their sole means of communication [3,4].

BCIs are broadly classified into invasive and non-invasive types. Invasive BCIs involve the implantation of electrodes within the cranium and offer high temporal and spatial resolution; however, they impose significant physical burdens due to the need for surgical procedures. Conversely, non-invasive EEG-based BCIs [5] have lower classification accuracy compared to their invasive counterparts, but their ease of application and suitability for daily use make them promising candidates for practical interface technologies [6,7,8].

EEG-based brain–computer interfaces (BCIs) can be broadly categorized into two types based on the characteristics of the brain activity they utilize: (1) those that rely on endogenous brain rhythms, and (2) those that rely on evoked potentials induced by external stimuli. The former includes motor imagery BCIs, which modulate sensorimotor rhythms through imagined movement, as well as passive BCIs that estimate the user’s attentional and emotional states [9,10,11]. The latter includes event-related potentials (ERPs), such as the P300 elicited by cognitive responses to external stimuli [12], as well as steady-state visual evoked potentials (SSVEP) [13,14] and code-modulated visual evoked potentials (cVEP) [15,16], which are elicited by visual stimulation. Among these, the P300-based BCI is one of the most practical and widely adopted non-invasive methods. It requires only three electrodes—a ground, a reference, and the Pz recording electrode—for operation. Moreover, it causes relatively little visual discomfort during prolonged use, thereby minimizing user burden. In addition, it provides sufficient classification accuracy, contributing to its advancement toward practical applications in non-invasive BCIs [17,18]. The P300 is a positive voltage component that appears approximately 300 ms after a subject is presented with and attends to a specific stimulus randomly presented among multiple visual or auditory stimuli at fixed intervals [19,20]. Leveraging this neurophysiological property, Farwell and Donchin (1988) developed a BCI system known as the “P300 speller”, which estimates the character a user is focusing on by displaying 26 letters on a screen and flashing them sequentially [21]. The impact of the P300 speller has been profound, such that a significant portion of non-invasive BCI research has since been dedicated to its refinement.

In prior studies aiming to enhance visual presentation methods for P300 detection, Kirasirova et al. reported that the flickering of surrounding characters in P300 spellers negatively affects classification accuracy. They demonstrated that restricting the visual field can emphasize the P300 peak waveform, thereby improving accuracy [22]. Kaufmann et al. achieved improved classification accuracy by replacing character stimuli with cognitively meaningful face images, which elicited more prominent P300 responses [23]. Furthermore, advancements have also been made in P300 signal analysis methods. Because the ERP-P300 component has a smaller amplitude than the background brain rhythms, conventional P300 analysis involves extracting EEG data within a fixed time window before and after sensory stimulation, applying baseline correction (adjusting the signal based on a stable reference voltage at a specific time point), and performing signal averaging to cancel out phase-incoherent background activity and reveal the P300 waveform [24,25,26,27]. However, in applications such as the P300 speller, where stimuli must be presented continuously at short intervals (e.g., 200 ms), maintaining EEG stability becomes challenging. As a result, identifying an appropriate baseline becomes difficult, and this baseline correction step may actually decrease P300 detection accuracy. To address this issue, Tanner and Norton et al. proposed a high-pass filter design capable of outputting a stable reference potential by simultaneously recording EEG and magnetoencephalography (MEG), with the aim of improving P300 detection performance [28]. Similarly, Krusienski et al. calculated a stable reference voltage based on EEG data from multiple electrodes and successfully demonstrated improved P300 detection rates [29]. Recently, machine learning-based P300 classification approaches have also been explored. Comparative evaluations have been conducted using classifiers such as Support Vector Machines (SVMs) and neural networks, including deep learning models [30]. Among these, studies using deep learning architectures such as EEG-Inception have significantly contributed to the generalization of P300 speller systems. Specifically, EEG-Inception has demonstrated the ability to detect P300 signals without requiring subject-specific calibration, which had previously been necessary for system operation [31]. Furthermore, research has also advanced in methods for detecting P300 by analyzing the temporal and spatial correlations of EEG signals measured from various brain regions using 8 to 32 electrode channels. In particular, a method has been developed that expresses the correlations between multiple channel signals using a covariance matrix and applies analysis based on Riemannian geometry [32], enabling high-accuracy P300 detection [33]. For example, Li et al. reported an AUC (area under the curve)—a machine learning performance metric ranging from 0 to 1, where 1 indicates perfect classification—of approximately 0.836, while Krzemiński et al. reported a classification performance of 81.2% based on accuracy [34,35].

Research on the practical implementation of the P300 speller is also progressing. For instance, Li et al. utilized a visual stimulus-based P300 speller to control a powered wheelchair [36], Piccione et al. applied a similar system for controlling a computer cursor [37], and Soram Kim et al. explored drone control within 3D virtual and augmented reality environments using visual stimulus-based P300 signals [38]. These studies collectively demonstrate that various external devices can be controlled based on the P300 speller framework.

However, such applications are not typically designed for welfare purposes and do not consider bedridden users, such as individuals with complete quadriplegia. In the case of bedridden users, visual stimuli for the P300 speller are primarily presented using display screens. It is crucial that these displays are positioned within the user’s visual field at an appropriate orientation. Consequently, every time the posture of the user is adjusted, the display must also be repositioned, thereby increasing the workload of caregivers. Furthermore, as the display is fixed directly in front of the user, their ability to visually perceive the surrounding environment is obstructed, negatively impacting their quality of life. Thus, there is a need for a novel visual stimulus presentation device that, unlike conventional displays, allows users to visually access their environment while still providing multiple visual stimuli regardless of changes in posture [39,40,41,42]. One prior attempt to address this issue is the table-type visual stimulus presentation device proposed by Mak et al. [43]. However, this approach merely reduces the display’s visible area by tilting the screen to expand the user’s view of the surrounding environment. As a result, the visual stimuli become harder to perceive, and the system cannot adequately accommodate changes in user posture. In recent years, P300-based BCIs that present visual stimuli via 3D virtual reality (VR) head-mounted displays (HMDs) have also been investigated [44]. These systems offer the advantage of being less susceptible to the effects of postural changes, as the display is worn directly on the user’s head. However, the weight and structure of HMDs may impose physical strain on the neck, and despite the ability to view the surrounding environment using 3D augmented reality (AR) functions, the continuous exposure to display light may lead to visual fatigue. Therefore, such systems may be unsuitable for prolonged use in bedridden individuals.

In this study, we propose a novel method for presenting visual stimuli in P300-based speller systems. Rather than displaying multiple stimuli on a single screen, we adopt a distributed approach, in which individual LED-based visual stimulus devices are wirelessly controlled and spatially arranged throughout the surrounding environment. This aims at realizing a P300 speller system based on wirelessly operated visual stimulus presentation devices. While wireless communication between the control system (e.g., PC) and EEG acquisition devices has been explored [45,46,47], there has been little prior research on wireless communication between the control system and the visual stimulus devices. This represents a novel direction. One of the main reasons for the lack of such studies is the widely accepted notion that precise timing of stimulus presentation is essential for P300 analysis [48]. Consequently, visual stimulus devices that involve wireless communication—which inherently introduces transmission delays—have not been utilized [49,50]. In conventional BCI studies, synchronization between stimulus presentation and EEG data acquisition is often achieved using wired connections via the Lab Streaming Layer (LSL) framework [51]. However, even with wired systems, timing delays are unavoidable. For instance, software-based detection of stimulus onset may suffer from irregular internal processing delays, and methods that detect visual stimulus timing using light sensors can be affected by inherent delays in the sensors themselves, often resulting in delays ranging from several to several tens of milliseconds [52]. To address this, researchers such as Andreev and Cattan have evaluated the impact of timing delays on ERP analysis [53,54], and Swami et al. highlighted the importance of delay correction in ERP analysis by measuring display onset delays using photodiodes [55]. Based on these findings, the present study aims to: (1) analyze the transmission delay characteristics of the proposed wireless visual stimulus system, (2) compare P300 detection performance between wired (zero-delay) and wireless (delay-included) stimulus presentation, and (3) propose and evaluate a method to improve P300 detection accuracy by estimating visual stimulus timing under wireless communication delay conditions.

In line with the above objectives, Section 2 details the proposed wireless visual stimulation devices along with the methodology for EEG measurement and P300 signal analysis. Section 3 presents Experiment 1, in which we conduct a quantitative analysis of the wireless transmission delay inherent to the proposed system and introduce a temporal correction method based on the delay characteristics. Section 4 reports Experiment 2, where P300 measurement and analysis were performed using the proposed device. In this experiment, the actual LED flash timing was recorded using a photodetector, and the voltage output was simultaneously captured with EEG signals, enabling the use of high-precision visual stimulus onset timing in the analysis. This setup allows for a comparison among three types of P300 detection methods: (1) detection based on photodetector-measured visual onset timings, (2) detection using visual onset timings with added delay modeled from Experiment 1, and (3) detection based on stimulus timings corrected using the proposed delay compensation method. A particular focus is placed on evaluating the waveform characteristics of the P300 response under each method. Section 5 presents Experiment 3, in which P300 measurement and analysis were conducted using the proposed device with 21 participants. This study provides a comprehensive evaluation of factors affecting P300 detection performance, including differences in viewing distances among devices, baseline correction timing, and the number of averaged trials.

## 2. Proposed System

### 2.1. Hardware Configuration

**Figure 1:**
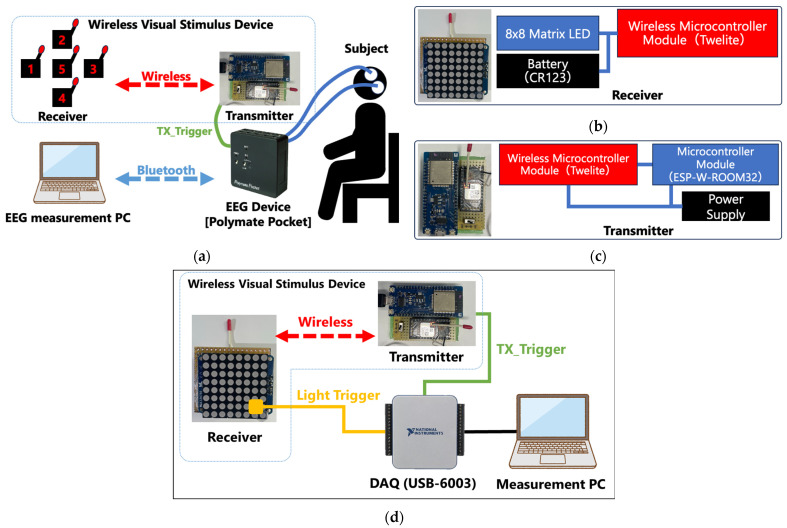
Hardware configuration: (**a**) the connection diagram of the measurement devices; (**b**) the receiver of the wireless visual stimulus presentation device; (**c**) the transmitter of the wireless visual stimulus presentation device; and (**d**) the auxiliary device for measuring the time difference between the transmission command and LED emission.

The hardware configuration of the proposed system, as shown in Figure 1a, consists of a Polymate Pocket EEG device manufactured by Miyuki Giken Co., Ltd. (Tokyo, Japan), a custom-developed wireless visual stimulus presentation device (comprising five receivers and one transmitter), and a note PC for measurement and analysis. The receiver of the wireless visual stimulus presentation device (Figure 1b) integrates a TWELite wireless communication module and an LED 8 × 8 dot matrix display, enabling the presentation of alphanumeric characters via LED illumination. The transmitter (Figure 1c) consists of a TWELite module and a microcontroller unit (ESP), and is responsible for controlling the random illumination of each visual stimulus presentation device at 600 ms intervals, as well as recording these illumination timings via external voltage input to the EEG device. However, because the illumination devices (receivers) and the illumination controller (transmitter) communicate wirelessly, temporal delays are introduced, which pose a significant issue compared to conventional display-based visual stimulus presentation methods. Therefore, in Experiments 1 and 2, an auxiliary measurement system was developed, as illustrated in Figure 1d, to measure the timing difference between when the transmitter sends a wireless command and when the LED on the receiver actually illuminates. Specifically, a USB−6001 data acquisition device by National Instruments was used. The timing of the wireless command is detected through the digital output voltage of the ESP microcontroller, while the actual illumination timing is determined from the voltage signal of a photodetector (S7184, Hamamatsu Photonics K.K., Shizuoka, Japan) attached to the LED matrix. This setup enables the precise calculation of the wireless communication delay. In addition, the timing of the LED emission and the output of the photodiode (S7184) were visually confirmed on an oscilloscope, verifying that the rising edges of both signals were aligned. To avoid the influence of ambient light during calibration, the area around the photodiode was covered with light-shielding tape so that only the LED light would reach the sensor.

### 2.2. Visual Stimulation Patterns

**Figure 2:**
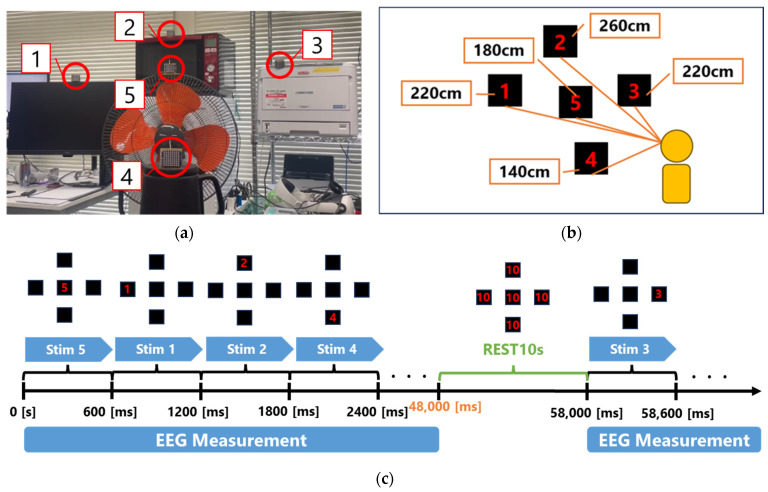
Visual stimulus presentation method: (**a**) the arrangement of the wireless visual stimulus presentation devices viewed from the front (numbers indicate stimulus identifiers); (**b**) the distances from the participant to the wireless visual stimulus presentation devices; and (**c**) the visual stimulus presentation protocol.

In this study, P300-evoked potential experiments were conducted based on five types of visual stimulation. As illustrated in Figure 2a,b, the wireless visual stimulus presentation devices were spatially arranged in a three-dimensional configuration. Unlike conventional monitor-based displays, each visual stimulus presentation device was placed at varying viewing distances from the participant: Stim1 at 220 cm, Stim2 at 260 cm, Stim3 at 220 cm, Stim4 at 140 cm, and Stim5 at 180 cm.

As shown in Figure 2c, the visual stimulation protocol involves randomly presenting each of the five stimuli 120 times during a single trial at 600 ms intervals, while ensuring that the same stimulus is not shown consecutively. To alleviate participant fatigue resulting from prolonged concentration, a 10 s break is provided after every 80 stimulus presentations.

### 2.3. EEG Measurement Settings

Electrode placement for EEG measurement followed the international 10–20 system. The ground (GND) electrode was placed on the forehead, the reference electrode on A1 (left earlobe), and the measurement electrodes on Cz and Pz, which are known to be effective for detecting P300 components. During electrode attachment, all electrode–skin impedances were kept below 30 kΩ to ensure proper signal quality. Participants were instructed to minimize head movements throughout the experiment. For each trial, they were directed to focus their gaze only on the specified visual stimulus target. Initially, the EEG signals passed through a 0.03 Hz high-pass filter implemented in the internal circuitry of the EEG device, followed by an anti-aliasing low-pass filter with a cutoff frequency of 333 Hz. The filtered signals were then recorded on a PC at a sampling rate of 1000 Hz. For subsequent analysis, a second-order Butterworth high-pass filter with a cutoff frequency of 0.05 Hz was applied in software. EEG signals were recorded from two channels (Cz and Pz), along with one channel for capturing the illumination timing signal from the wireless visual stimulus transmitter. Because this study was intended for welfare applications, the experiments were conducted not in an electromagnetically shielded room, but in a standard room simulating a typical home or office environment. Care was taken in the arrangement of electronic devices powered by commercial electricity to prevent the generation of power line noise. Fluorescent lighting commonly used in Japanese offices was employed, and sunlight was blocked to avoid direct exposure. In addition, strong spotlights were not used to prevent interference with the visual stimulus presentation.

### 2.4. P300 Analysis Method

**Figure 3:**
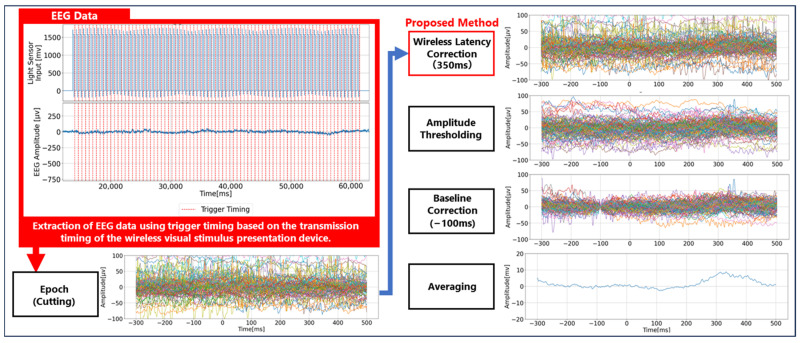
The process of calculating the P300 peak from EEG data. The colored lines represent EEG segments extracted using trigger timings based on the transmission timings of the wireless visual stimulus presentation device.

The algorithm for analyzing EEG event-related potentials (ERPs), specifically the P300 component, was implemented using Python (version 3.12.7) on the measurement and analysis PC. As illustrated in Figure 3, the analysis procedure begins by extracting EEG data within a 1000 ms window centered around the flash command timing obtained from the transmitter of the wireless visual stimulus presentation device. These segments are then categorized according to each visual stimulus. Next, a temporal correction is applied to account for wireless communication delays, allowing the accurate estimation of the actual visual stimulus onset. Based on this corrected timing, a 500 ms segment before and after the visual stimulus is extracted. Trials in which the maximum EEG amplitude exceeds ±100 µV are automatically excluded, as such extreme values are considered to reflect artifacts such as eye movements or body motion, which can interfere with P300 detection. Following artifact rejection, baseline correction is applied using the amplitude at 100 ms prior to the stimulus onset. Subsequently, averaged waveforms are computed across trials to suppress background rhythms and enhance the signal-to-noise ratio, enabling the detection of the subtle ERP waveform known as the P300, which typically has an amplitude of approximately +5 μV. Among the averaged EEG waveforms classified by the five visual stimuli, the one exhibiting the largest positive peak within the 200–400 ms window post-stimulus is considered to correspond to the target stimulus that the participant was focusing on. Conversely, visual stimuli for which no P300 peak is detected are classified as non-target stimuli. This study is aimed at improving convenience in welfare applications, and therefore emphasizes minimizing the number of electrodes required for operation. To this end, we focus on the Pz electrode, where the P300 component is most prominently observed, and conduct signal analysis solely based on the P300 waveform recorded at this site. Consequently, common preprocessing techniques that require multiple channels—such as re-referencing, channel interpolation or rejection, and artifact removal methods including ICA and ASR—are not applied in this study.

## 3. Experiment 1: Evaluation of Wireless Transmission Delay in Visual Stimulus Presentation Device

Because the proposed wireless visual stimulus presentation device introduces latency due to wireless communication, the accurate estimation of the actual visual stimulus onset time was crucial for reliable P300 analysis. Therefore, in Experiment 1, the characteristics of the wireless transmission delay inherent to the system were analyzed. Based on the results, a methodology for estimating the actual stimulus presentation timing—referred to as the temporal correction method—was examined to support P300 signal processing.

### 3.1. Experimental Setup for Evaluation of Wireless Transmission Delay

Using the auxiliary measurement system described in Section 2.1, measurements were conducted to assess the timing difference between the wireless command issued by the transmitter and the actual LED illumination detected by the photodetector on the receiver. These measurements were performed under three different transmitter-to-receiver distance conditions: 1 m, 2 m, and 3 m. The transmitter sends periodic wireless signals consisting of 200 ms rectangular pulses. A single pulse functions as a toggle command: if the LED is off, it is instructed to turn on; if it is on, it is instructed to turn off. Thus, the LED operates in a cycle—turning on, remaining illuminated for 500 ms, turning off, and then turning on again after a 1000 ms interval—driven by the periodic wireless command.

### 3.2. Experimental Results for Evaluation of Wireless Transmission Delay

**Figure 4:**
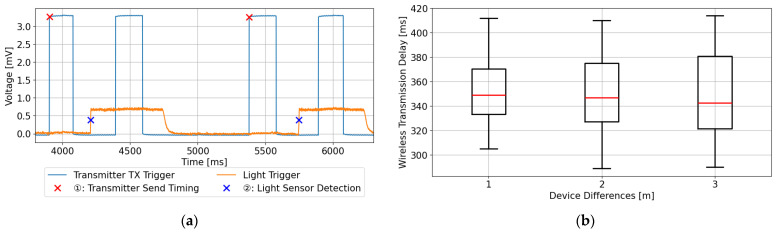
Experimental results: (**a**) the voltage waveforms of the LED emission command before wireless transmission and the actual LED emission detected by the optical sensor, and (**b**) the statistical analysis of wireless transmission delays under each distance condition (1 m/2 m/3 m). The red line in each box plot indicates the median delay.

Figure 4a shows the voltage waveform corresponding to the LED flash command issued via wireless communication, along with the voltage waveform obtained from the photodetector that captures the actual LED illumination state. The red X markers indicate the intended flash command timing, while the blue X markers denote the actual visual stimulus onset as detected by the sensor. As shown, a transmission delay is observed between the command and the actual LED activation, confirming the presence of wireless communication latency.

The statistical results of transmission delay measured at three different distances (1 m, 2 m, and 3 m) are presented in Figure 4b. The median delay times were 349 ms at 1 m, 347 ms at 2 m, and 342.5 ms at 3 m. The average delay times were 353.5 ms (1 m), 350.2 ms (2 m), and 352.5 ms (3 m), with an overall mean of 352.1 ms. In all cases, no notable change in average delay was observed with respect to distance. However, focusing on the variability of the delay, a tendency of increasing dispersion with greater distances was confirmed. Therefore, we propose a temporal correction method in which a fixed value of 350 ms is added to the flash command timing to estimate the actual visual stimulus onset. The average standard deviation across 1–3 m was 30.9 ms.

## 4. Experiment 2: Evaluation of Waveform Characteristics Based on P300 Experiments Using Proposed Wireless Visual Stimulus Presentation Device

### 4.1. Experimental Setup for Evaluation of Waveform Characteristics

To evaluate the effects of wireless transmission delay on P300 waveform characteristics and detection performance, a P300 detection experiment was conducted using the proposed wireless visual stimulus presentation device—which introduces communication latency—and the auxiliary measurement system capable of accurately capturing visual stimulus presentation timings. The experiment was carried out with one participant and consisted of two trials, each employing a different visual stimulus as the target stimulus. To quantitatively assess the influence of transmission delay on P300 waveforms, three different analytical methods were employed for P300 signal extraction:

1. Transmission signal-based corrected (TSC) method: a method using the proposed temporal correction from Experiment 1, where a fixed delay of 350 ms is added to the flash command timing to estimate stimulus onset.
2. Actual visual onset-based (AVO) method: an ideal method using the actual visual onset time directly measured by the photodetector, serving as a reference for accurate P300 timing.
3. Jitter-modeled correction (JMC) method: a method combining the measured visual onset time with a modeled delay distribution derived from Experiment 1, where random delays sampled from a Gaussian distribution (mean: 352.1 ms, standard deviation: 30.9 ms) are added to simulate real-world wireless latency characteristics.

By calculating P300 waveforms using these three approaches and comparing them, this experiment investigated how transmission delay and its variability affect the accuracy and reliability of P300 detection. In the JMC method, pseudo-random delays were generated based on a normal distribution using the wireless transmission delay characteristics obtained in Experiment 1 (mean: 352.1 ms, standard deviation: 30.9 ms) and were constrained within a range of 280 to 420 ms.

### 4.2. Experimental Results for Evaluation of Waveform Characteristics

**Figure 5:**
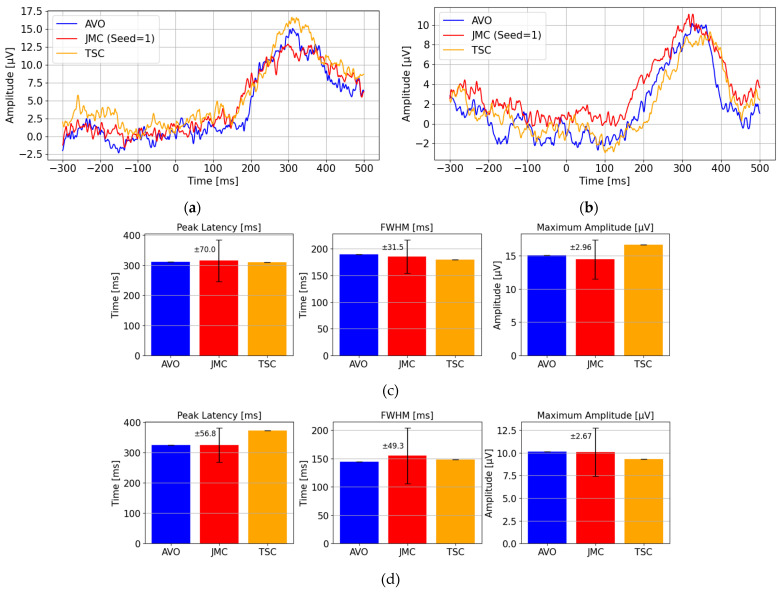
Comparison of P300 waveforms across AVO, JMC, and TSC: (**a**) Comparison of P300 waveforms obtained by averaging 40 trials for Target Stimulus 3; (**b**) comparison of P300 waveforms obtained by averaging 40 trials for Target Stimulus 5; (**c**) bar graphs with error bars showing the mean and standard deviation of peak time, FWHM, and max amplitude for Target Stimulus 3; (**d**) bar graphs with error bars showing the mean and standard deviation of Peak Time, FWHM, and max amplitude for Target Stimulus 5. The error bars for JMC represent ±3 standard deviations (±3σ) based on 100 simulated P300 analyses, whereas AVO and TSC are based on single-trial data, and thus no error bars are displayed for them.

The P300 analysis results obtained using the three methods were evaluated over two trials, with Stimulus 3 and Stimulus 5 designated as the target stimuli. These results are shown in Figure 5a,b. With all three methods, similar positive peak waveforms were observed around 300 milliseconds after the visual stimulus, indicating that P300 detection was feasible regardless of the analytical approach. However, the TSC and JMC methods are inherently affected by the variability introduced by wireless transmission delays. Therefore, a comparative analysis was conducted across the three methods focusing on three key metrics (Figure 5c,d):

1. The peak latency (the time of maximum amplitude);
2. The full width at half-maximum (FWHM) of the peak;
3. The maximum amplitude.

In particular, the JMC method introduces stochastic variation based on the transmission delay characteristics identified in Experiment 1. Using these characteristics (mean: 352.1 ms, standard deviation: 30.9 ms), 100 simulated P300 analyses were performed, and the resulting standard deviations were used to quantitatively evaluate the impact of wireless delay on P300 waveform morphology. The error bars in the figures represent ±3 standard deviations (±3σ), visually illustrating the expected range of variation due to wireless communication delays.

The analysis of the JMC (Joint Morphological Characteristics) results indicates that the temporal variability introduced by wireless communication delay was adequately captured. The peak latency falls within the range of 240 ms to 380 ms, and the maximum amplitude ranges within ±3.0 μV. Furthermore, FWHM demonstrates that the fundamental characteristics of the original P300 waveform were preserved. Based on these findings, we conclude that despite the presence of wireless-induced transmission delays, the resulting waveform still supports reliable P300 detection.

## 5. Experiment 3: Evaluation of P300 Detection Using Wireless Visual Stimulus Presentation Device in 21 Participants

### 5.1. Experimental Setup for Evaluation of P300 Detection

In the previous sections, it was demonstrated that P300 detection is feasible when using the proposed wireless visual stimulus presentation device, provided a temporal correction of 350 ms is applied to account for wireless transmission delay. In this experiment, P300 detection trials were conducted on 21 participants using the proposed system. Each participant underwent two trials, with Stimulus 3 and Stimulus 5 designated as the target stimuli. Unlike traditional display-based visual stimulation systems, the proposed system presents stimuli at varying viewing distances, which may result in differences in visual stimulus intensity. Accordingly, the influence of such variations on P300 detection performance was also examined.

### 5.2. Experimental Results for Evaluation of P300 Detection

**Figure 6:**
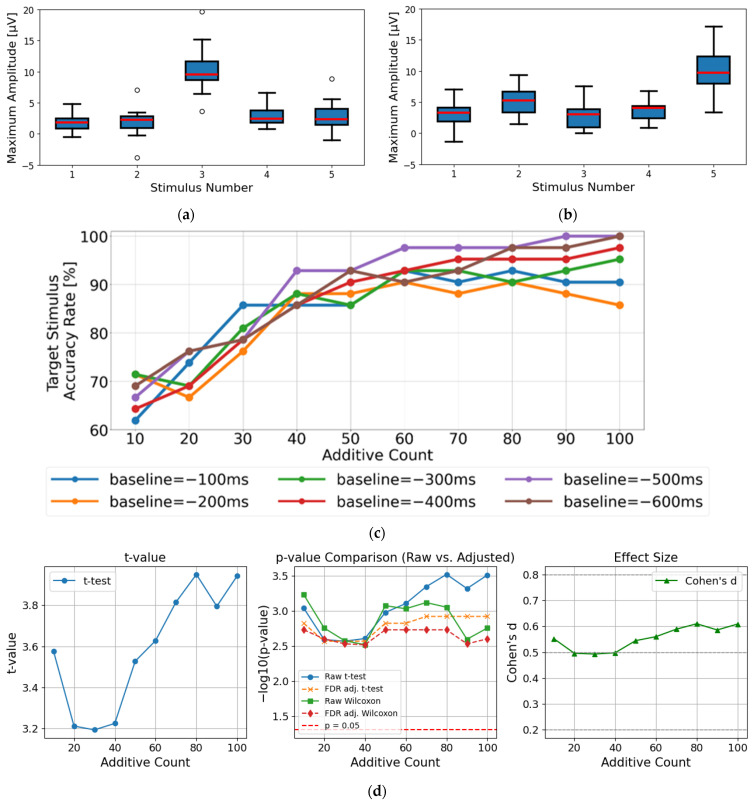
The evaluation of P300 detection under the transmission signal-based corrected (TSC) condition: (**a**) the comparison of maximum amplitude values for each stimulus under Target Stimulus 3 (red lines indicate medians; circles represent outliers); (**b**) the comparison of maximum amplitude values for each stimulus under Target Stimulus 5 (red lines indicate medians); and (**c**) the detection rate of the target stimulus, defined as the percentage of trials (*n* = 42; 21 participants × 2 trials) in which the target stimulus showed the highest amplitude within the 200–400 ms evaluation window. (**d**) Statistical comparison of maximum amplitude values between target and non-target stimuli as a function of the number of averaging trials. The left panel shows t-values, the center panel shows *p*-values (−log10 transformed), and the right panel shows the effect size (Cohen’s d).

Figure 6a,b present the statistical results of the maximum amplitude values for each visual stimulus, calculated from the EEG data averaged over 100 trials per participant. In both trials, clear distinctions between target and non-target stimuli were observed, demonstrating the effectiveness of the proposed wireless visual stimulation system for P300 detection. These results indicate that LED-based stimulation is sufficient to evoke P300 responses, confirming that the system provides adequate visual stimulus intensity despite variability in viewing distance.

Furthermore, Figure 6c illustrates the P300 detection rates across all 42 trials (21 participants × 2 trials), with respect to varying numbers of averaging repetitions and different baseline periods (−100 ms, −200 ms, −300 ms, −500 ms, and −600 ms). When the number of averaging trials was 40 or more, all baseline conditions achieved high classification accuracy, exceeding 80%. Among them, conditions using baseline points at −500 ms and −600 ms consistently exhibited higher accuracy compared to other baselines. In particular, the −500 ms baseline achieved the highest classification accuracy in the 40–100 trial range, while the −600 ms baseline showed peak performance around 80–100 trials. These results indicate that the −500 ms to −600 ms range is the most effective baseline period. Given that the visual stimuli were presented at 600 ms intervals, and the wireless transmission delay exhibited a mean of 352.1 ms with a standard deviation of 30.9 ms. These findings suggest that the optimal baseline window shifts accordingly. Therefore, the period 500–600 ms prior to stimulus onset is considered effective for baseline referencing.

Figure 6d shows the statistical test results comparing the maximum amplitude differences between target and non-target stimuli across different averaging conditions. In all conditions with 10 or more averaging trials, both paired *t*-tests and Wilcoxon signed-rank tests revealed statistically significant differences (*p* < 0.01). To account for potential false positives due to multiple comparisons, false discovery rate (FDR) correction using the Benjamini–Hochberg method was applied to the obtained *p*-values. The results remained statistically significant after FDR correction in all conditions (e.g., for 10 trials: t = 3.58, raw *p* = 0.0009, FDR-corrected *p* = 0.0015), indicating that the observed effects were not due to chance. The effect sizes (Cohen’s d) were moderate across all conditions (approximately 0.49 to 0.61), with slightly larger effects observed at 80 and 100 averaging trials (around 0.6). These results confirm that the proposed wireless visual stimulus system is effective for eliciting significantly detectable P300 components.

**Figure 7:**
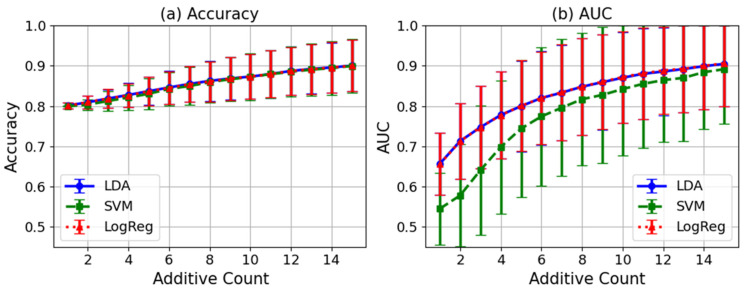
Comparison of classification performance using three classifiers (LDA, SVM, and Logistic Regression) for P300 detection based on EEG features from Cz and Pz channels. (**a**) Mean and standard deviation of classification accuracy for each number of averaging trials; (**b**) Mean and standard deviation of AUC (area under the curve) for each number of averaging trials.

To further evaluate the classification performance of P300 waveforms on a per-trial basis, a comparative analysis was conducted using three classifiers—Linear Discriminant Analysis (LDA), Support Vector Machine (SVM), and Logistic Regression (LogReg)—based on features extracted from the Cz and Pz channels. Figure 7 presents the mean and standard deviation of classification accuracy and area under the curve (AUC) for 21 participants at each level of averaging.

For classifier evaluation, EEG data from the Cz and Pz channels were collected for each of the 21 participants, with 100 trials per stimulus. In the averaging process, a fixed number of consecutive trials (defined by the averaging count) were averaged, and this averaging was repeated while shifting the starting point by one trial at a time [29]. For example, when the averaging count was 15, a total of 85 averaged segments were generated from 100 trials. This procedure was applied for each averaging count from 1 to 15, and the mean amplitude in the 200–400 ms post-stimulus window was used as a feature. Target and non-target labels were assigned to each sample, and binary classification was performed. LDA, SVM, and Logistic Regression were used as classifiers, and classification accuracy and AUC were calculated. These classifiers are widely used in previous studies on single-trial P300 classification. For example, LDA is frequently employed in P300 classification due to its simplicity and stable performance [56,57], while SVM has been reported to exhibit high generalization performance as a linear classifier for EEG classification [58,59]. Logistic Regression has also been demonstrated to improve single-trial classification performance in P300 speller systems [60,61]. Regarding classifier settings, default parameters were used for LDA; for SVM, a linear kernel with class_weight = ‘balanced’ and probability = True was adopted. For Logistic Regression, the maximum number of iterations was set to 1000, with other parameters kept at default. The selection of classifiers and feature extraction methods was based on standard frameworks presented in previous studies [62,63]. Training and evaluation of the classifiers were conducted independently for each participant, and separate models were built for individual participant data. Since practical BCI systems require a balance between response time and detection performance, and prior studies have reported high performance with averaging over 10–20 trials [64,65], the maximum averaging count was set to 15. For cross-validation, trials were treated as independent units, and leave-one-group-out cross-validation was applied to ensure that data from the same trial did not overlap between training and evaluation sets [62,66].

As shown in Figure 7a, accuracy exhibited an increasing trend with the number of averaged trials for all classifiers, reaching an average accuracy of approximately 0.90 at 15-trial averaging for LDA, SVM, and LogReg. Regarding AUC, as shown in Figure 7b, Logistic Regression consistently outperformed the other classifiers, with its AUC increasing with the number of averaged trials and reaching around 0.91. On the other hand, SVM exhibited greater variability in classification performance, with standard deviations higher than those of the other classifiers. These results confirm that reliable classification performance can be achieved for P300 detection using features from the Cz and Pz channels.

## 6. Discussion

In this study, we validated the effectiveness of the proposed system using a wireless visual stimulus presentation device for P300 detection, focusing on baseline correction timing and classifier performance. The following sections present a discussion of the results from each perspective.

### 6.1. Validity of Baseline Correction Timing at −500 ms/−600 ms

**Figure 8:**
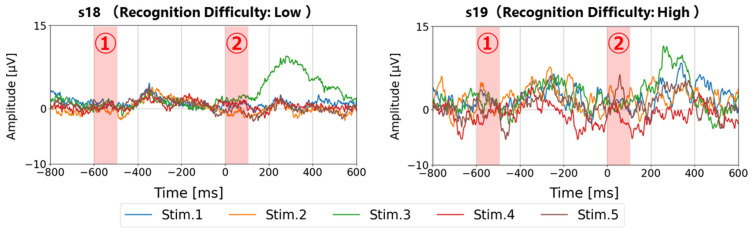
Example comparison of all-stimulus waveforms (100-trial averaging) for participants S18 and S19 (baseline: −500 ms; target stimulus: Stim.3). Red box ① indicates the interval from 500 to 600 ms before stimulus onset, and red box ② indicates the interval from 0 to 100 ms after stimulus onset.

A comparative evaluation was conducted on the detection performance of P300 based on six types of single-point amplitude baselining intervals within 100 ms before visual stimulus onset (i.e., at −100 ms, −200 ms, −300 ms, −400 ms, −500 ms, and −600 ms). The results showed that baseline corrections using the −500 ms and −600 ms time points yielded the highest P300 detection rates. This is considered to be because, in the interval from 0 to 500 ms before the visual stimulus, P300 is elicited in response to target stimuli, and event-related potentials such as N200 may occur in response to stimulus interval perception even for non-target stimuli, whereas the interval from 500 ms to 600 ms before the stimulus tends to remain stable. As shown in Figure 8, section ① (the 500–600 ms pre-stimulus interval) corresponds to the 0–100 ms post-stimulus interval of the previous trial, where N100/P100 waveforms are not observed, thus providing a stable EEG reference potential. This is also supported by the fact that section ② in Figure 8 (0–100 ms after the visual stimulus) shows more stable amplitude than the periods after 100 ms. The suppression of N100/P100 components may be due to phase cancelation effects caused by the ±30.9 ms jitter in wireless communication delay, as well as the use of a high-pass filter at 0.05 Hz, both of which may reduce amplitude. Ultimately, it was confirmed that identifying a stable EEG baseline in accordance with system-specific parameters—such as the deviation characteristics of wireless communication delay (±30.9 ms), inter-stimulus interval (600 ms), and high-pass filter setting (0.05 Hz)—is crucial for determining the optimal baseline correction timing.

### 6.2. Effects of Ambient Lighting and Visual Perception

In this study, the lighting environment was meticulously controlled under conditions simulating a typical office setting. Specifically, direct exposure to natural sunlight was blocked, and the use of high-intensity spot lighting was avoided in order to minimize interference from ambient light during visual stimulus presentation. Visual stimuli were delivered using wirelessly controlled LED devices, which produced distinct and consistent luminance changes via blinking patterns, enabling clear discrimination from background illumination. Consequently, the influence of environmental lighting on stimulus perception is presumed to be minimal. Furthermore, the employed LED devices were structurally designed to allow adjustment of emission color, providing the potential for future extension toward dynamic optimization of stimulus color and intensity according to individual subject characteristics and specific experimental environments.

**Figure 9:**
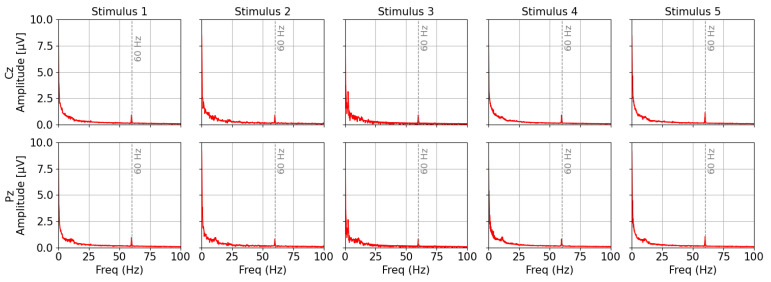
FFT amplitude spectra of Cz and Pz channels (average of 21 participants; the dashed line indicates the 60 Hz frequency of power-line noise).

As a preliminary evaluation prior to the main experiment, FFT spectral analysis was conducted on the Cz and Pz channels to assess the quality of the EEG data. Figure 9 shows the averaged FFT spectra over two trials from 21 participants. The analysis revealed a minor noise peak around 60 Hz, which is attributable to power-line interference, with an amplitude of approximately 1 µV. However, this amplitude is very small compared to the overall spectral amplitude range of the EEG signals (below 10 µV), and thus its impact on ERP analysis is considered negligible [27,67]. In addition, no prominent peaks were observed in the high-frequency band above 30 Hz, which is typically associated with electromyographic artifacts [68].

### 6.3. Influence of Wireless Transmission Delay on Classification Performance and Real-Time Applicability

In this study, P300 detection was performed using classifiers based on LDA, SVM, and Logistic Regression. Notably, both LDA and Logistic Regression achieved an average accuracy of 0.80 and an AUC of 0.66 even in single-trial conditions, where no averaging was applied to the EEG data for each stimulus. The features used were limited to the mean amplitude in the 200–400 ms post-stimulus window and only two channels (Cz and Pz), demonstrating that a simple configuration can still yield a certain level of classification performance. However, the observed AUC was slightly lower compared to previously reported single-trial classification results in existing P300 BCI studies (0.75–0.85) [62,65]. This may be partly attributed to the temporal variability (jitter) in stimulus presentation timing caused by wireless communication. As revealed in Experiment 1, wireless transmission exhibited an average delay of 352.1 ms with a standard deviation of 30.9 ms. Such temporal jitter can shift the P300 peak position across trials, reducing waveform amplitude during averaging and consequently degrading classification performance [25].

To address the variability in P300 waveforms caused by stimulus timing jitter, Congedo et al. (2016) proposed a jitter correction method based on covariance maximization, known as the Spatio-Temporal Common Pattern [69]. This method temporally aligns each trial’s waveform to an ideal template waveform (obtained through sufficient averaging that enhances the P300 component) by applying the time shift that yields the greatest similarity. By correcting the temporal misalignment of P300 peaks across trials, the method enables classification without trial averaging, mitigating amplitude attenuation and performance loss. It is also applicable to single-channel configurations and, when combined with lightweight classifiers, is expected to be a practical real-time single-trial classification approach suitable for welfare-oriented and simplified BCI applications. In the present study, it is anticipated that this template-based jitter correction could be applied in addition to the fixed latency correction (350 ms) already implemented for wireless delays, thereby compensating for residual variations in visual stimulus timing and further improving classification performance in wireless environments.

Furthermore, recent studies suggest that an AUC of 0.75 or higher is desirable for reliable single-trial P300 classification [70]. From this perspective, the AUC of 0.66 obtained in this study indicates room for improvement. Other approaches have achieved high single-trial classification performance—AUCs exceeding 0.90 and accuracies near 90%—by utilizing multiple channels to exploit spatial information from electrode configurations in conjunction with machine learning techniques [30,31]. Incorporating such methods into the current approach is expected to yield further performance gains. In addition, the Mother of All BCI Benchmarks (MOABB) framework has been developed to evaluate single-trial classification performance across multiple public datasets, aiming to standardize classification performance and ensure reproducibility in BCI research [71]. For P300 tasks, high-performance classification results with AUCs exceeding 90% have been reported using pipelines such as xDAWNCov + TS + SVM across various datasets [72].

On the other hand, under conditions in which 10 to 15 trials were averaged, the classification accuracy reached 0.85–0.90, which is comparable to the performance levels reported for conventional display-based P300 speller systems [64,65]. This result demonstrates that stable P300 detection is achievable even under wireless jitter constraints, provided that appropriate averaging is applied.

It should be noted that the objective of this study is to construct a practical P300 detection system for welfare applications. Therefore, instead of using shielded environments designed to minimize noise for high-precision neuroscientific studies, EEG measurements were conducted in an unshielded office setting where commercial noise is present. To reduce user burden, a low-channel setup (four electrodes) was used, and only trials with large artifacts (e.g., ocular or movement artifacts exceeding ±100 μV) were excluded. The primary aim was to examine whether a scalp EEG-based interface could still be functionally viable even if P300 waveforms were degraded by wireless transmission. Accordingly, detailed analysis of how continuous stimuli affect P300 waveform distortion, as well as enhancement of classification accuracy through advanced signal processing techniques such as wavelet transform, empirical mode decomposition (EMD), independent component analysis (ICA), and Kalman filter-based methods, as proposed in previous studies [73,74,75,76,77], are considered important future work.

In conclusion, although challenges remain for real-time single-trial classification, the simplicity of the classifiers used and the lightweight nature of the artifact rejection method suggest that, when combined with appropriate averaging, the proposed system holds sufficient potential for implementation as a practical BCI system designed for welfare applications.

### 6.4. Limitations and Future Challenges

This study confirmed that P300 detection is feasible even under the presence of transmission delays inherent in the wireless visual stimulus presentation system. However, it was also observed that such delays contribute to a reduction in detection accuracy. In the present implementation, a fixed time correction of 350 ms was applied to compensate for the wireless transmission delay. Nevertheless, the variation (i.e., jitter characteristics) of the delay was not fully accounted for, which is presumed to be a major factor underlying the reduction in P300 detection performance. These jitter characteristics cause fluctuations in the actual stimulus onset timing, resulting in temporal misalignment of the P300 peak latency. Consequently, this leads to a degradation in the maximum amplitude of the averaged P300 waveform due to the temporal dispersion of individual responses during signal averaging. To address this issue, a future enhancement would involve ranking candidate target stimuli—based on the method developed in this study—and focusing on the top three stimuli likely to elicit a P300 response. For these stimuli, the pre-averaged EEG segments would be individually analyzed to assess residual rhythmic activity and waveform morphology. If temporal misalignment in the P300 peak is identified, realignment procedures based on the peak amplitude latency could be implemented in an iterative manner to refine the accuracy of P300 detection. It is worth noting that the issue of EEG baseline correction—another critical component in P300 analysis—has been resolved in this study. The −500 ms to −600 ms interval prior to stimulus onset was identified as a stable EEG reference baseline. In summary, the primary remaining challenge for improving P300 detection performance in this system lies in the compensation of jitter characteristics associated with wireless transmission latency.

Finally, this study presented a case demonstrating the feasibility of P300 detection using a wireless visual stimulus presentation system with 600 ms stimulus intervals, developed under the premise of reducing user burden. It also exemplified the basic applicability of such a system to scalp EEG-based brain–computer interfaces. Furthermore, even with machine learning-based classification, the system was shown to be capable of detecting P300 to a minimum acceptable level when compared with previous studies. However, as this is only a single case study, future work must include neuroscientific validation using a multi-electrode setup and measurements in a shielded environment. Planned studies will also include detailed analysis of P300 waveform changes across different stimulus intervals and evaluation of relationships with signal processing methods used in previous studies, including the optimization of machine learning parameters.

## 7. Conclusions

This study aimed to enhance the usability of the P300 speller system, which evokes the P300 event-related potential through multiple visual stimuli presented on a display, for the purpose of enabling individuals with complete paralysis to operate external devices. Conventional P300 speller systems rely on monitor-based visual stimulus presentation, which poses practical challenges for bedridden individuals—such as the need to precisely reposition the display for each session and the obstruction of peripheral vision—making it difficult to observe the surrounding environment. To address these issues, we proposed a wireless visual stimulus presentation device that can be flexibly and spatially distributed throughout the surrounding environment. However, due to the inherent variability in wireless communication delays, such devices have traditionally been considered unsuitable for P300 analysis, and prior studies in this domain have been lacking. In Experiment 1, we quantitatively analyzed the characteristics of wireless transmission delay in the proposed system (mean: 352.1 ms, standard deviation: 30.9 ms) and proposed a 350 ms temporal correction method to estimate the actual timing of visual stimulus onset for accurate P300 analysis. In Experiment 2, we developed an auxiliary measurement device capable of recording both the command timing and the actual LED onset timing of the receiver. A P300 elicitation experiment with one participant was conducted, and the influence of wireless jitter on the peak latency, full width at half maximum (FWHM) of the P300 waveform, and maximum amplitude was quantitatively simulated. The results confirmed that, despite wireless delay, the P300 waveform remained detectable. By applying the delay model to EEG data segments aligned with actual LED onset times, we generated virtual delay-affected datasets and successfully demonstrated the impact of delay on P300 waveform characteristics. Despite the influence of delay, reliable P300 detection remained feasible. In Experiment 3, P300 evocation trials using the proposed wireless stimulation device were conducted on 21 participants, each performing two trials with different target stimuli. In both cases, clear distinctions in the maximum amplitude between target and non-target stimuli were observed, confirming the effectiveness of the system. Furthermore, while conventional practice recommends baseline correction using the 100 ms pre-stimulus period, our results revealed that setting the baseline at 500–600 ms before the stimulus onset yielded the highest detection rates, particularly under conditions with a 600 ms stimulus interval and wireless delay characteristics (mean: 352.1 ms, standard deviation: 30.9 ms). In summary, this study demonstrates that, even with wireless visual stimulus delivery, stable and reliable P300 detection is achievable by applying a 350 ms temporal correction and setting the baseline at 500–600 ms prior to the visual stimulus. These findings support the feasibility of a spatially distributed, display-free P300 interface system suitable for individuals with severe physical impairments.

## Data Availability

The EEG data presented in this study are not publicly available due to privacy and ethical restrictions.
